# Case Report: Glucocorticoids Combined With Immunosuppressant in the Treatment of Acromegaly Complicated With Focal Segmental Glomerulosclerosis

**DOI:** 10.3389/fmed.2020.563020

**Published:** 2021-01-15

**Authors:** Ruiqiang Wang, Yunqi Wu, Dongyue An, Pupu Ma, Yuanyuan Guo, Lin Tang

**Affiliations:** Department of Nephrology, The First Affiliated Hospital of Zhengzhou University, Zhengzhou, China

**Keywords:** acromegaly, focal segmental glomerulosclerosis, growth hormone, glucocorticoids, immunosuppressant

## Abstract

**Background:** Acromegaly is a chronic disease caused by excessive secretion of growth hormone (GH), which circulates and stimulates the liver and body tissues to produce insulin-like growth factor type 1 (IGF-1). Experimental studies have shown that excessive secretion of GH is related to glomerular sclerosis, and elevated IGF-1 levels may be involved in the occurrence of glomerular hypertrophy. But relevant clinical cases are rare. Here, we reported a case of acromegaly complicated with focal segmental glomerulosclerosis (FSGS).

**Case Presentation:** A 49-year-old man was admitted to our hospital because of acromegaly for more than 10 years and proteinuria for more than 3 years. Acromegaly was confirmed by contrast-enhanced magnetic resonance imaging, minimally invasive surgery and pathology. The results of renal biopsy showed FSGS-NOS (not otherwise specified) with ischemic renal injury and mesangial IgA deposition. One month after transnasal transsphenoidal space occupying resection, GH and urinary protein decreased significantly, and nephropathy was partially relieved. In the next 4 months, GH stabilized at the normal level, while urinary protein gradually increased. When the urinary protein increased to 4.2 g/d, the dosage of glucocorticoids increased to 20 mg/d, and tacrolimus 1 mg/d were added, and the urinary protein decreased again. However, when the urinary protein decreased to 0.43 g/d, the patient stopped taking glucocorticoids and tacrolimus, and the urinary protein increased to 2.85 g/d after 8 months, but the GH was still in the normal range.

**Conclusion:** In this case, GH is partially involved in the formation of FSGS. Not only does surgery reduce the effects of GH, but low doses of glucocorticoids and immunosuppressant are effective in slowing the progression of kidney disease, at least in reducing urinary protein.

## Background

Acromegaly is a slowly progressive disease due to increased release of growth hormone (GH) and insulin-like growth factor type 1 (IGF-1). Most cases are caused by pituitary adenomas secreting GH (1). Excessive secretion of GH leads to excessive secretion of IGF-1, which affects the structure and function of each body system (2). Focal segmental glomerulosclerosis (FSGS) is a histological lesion caused by glomerular injury that mainly affects podocytes, including immune-mediated and other causes of podocyte damage, characterized by the presence of sclerosis in parts (segmental) of some (focal) glomerulus (3). In animal experiments, it has been found that excessive secretion of GH mediates podocyte damage (4), causing glomerulosclerosis and proteinuria (5), while abnormally high IGF-1 can lead to glomerular hypertrophy (6). Herein, we present a rare case of acromegaly with FSGS, emphasize the importance of clinical and histological presentation of this rare condition, concise review of treatment options, and analyze the possible relationship between acromegaly and FSGS.

## Case Presentation

A 49-year-old man was hospitalized with a one-and-a-half-year history of abnormal urine test and elevated blood sugar in January 2018. Three and a half years ago, it was found that the urinary protein was 2+, the fasting blood glucose was more than 20 mmol/L, and the blood pressure was 150/95 mmHg. There were no obvious concomitant symptoms. Two and a half years ago, he developed proteinuria 3+, fasting blood glucose was more than 20 mmol/L, blood pressure (BP) was 140/95 mmHg, accompanied with a lot of foamy urine, without gross hematuria, increased nocturia, polydipsia, polyphagia and weight loss, and received a treatment with “nifedipine and metformin.” 2 years ago, the 24-h urinary protein (24 h-TP) was 4.88 g, serum albumin (ALB) 41 g/L, no visual field changes, no joint pain, and irregular oral medication. Past history: Acromegaly had a history of more than 10 years, nasal hypertrophy had a history of 3 years, and diabetes mellitus (DM) has a history of more than 2 years. Family history: There was no similar disease or family history.

**Figure 1:**
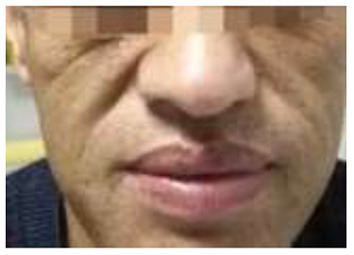
Facial manifestations of acromegaly.

At admission, the patient had a body temperature of 36.5°C, blood pressure of 154/86 mmHg, height of 180 cm, weight of 87 kg and the body mass index of 26.9 kg/m^2^. Physical examination showed evidences of acromegaly: Acral overgrowth of the hands and feet, frontal bossing, and enlargement of the tongue and lips (Figure 1). The heart had a regular rate and rhythm without rubs, gallop or murmurs. Breathing sounds were not vesicular and no murmurs were audible. No abnormalities were observed during the abdominal examinations. No percussion pain in renal area. There was no edema in both lower limbs. Physiological reflex existed while pathological reflex was not elicited.

Laboratory examination results were as follows: white blood cell count, 5.9 × 10^9^/L; red blood cell count, 5.27 × 10^12^/L; hemoglobin, 154 g/L; platelet count, 208 × 10^9^/L; proteinuria, 2+; urine sediments, 3 red blood cells per high-power field; 24h-TP, 6.29 g; blood urea nitrogen, 4.8 mmol/L; serum creatinine (SCr), 61 μmol/L; estimated glomerular filtration rate (eGFR), 111.467 mL/min/1.72 m^2^*;* ALB, 37.1 g/L; glycosylated hemoglobin, 6.9%; ESR, 21 mm/hr; CRP, 0.58 mg/L; comlement C3, 0.96 g/L; comlement C4, 0.24 g/L; GH, 26.4 ng/mL; IGF-1, 1,108 ng/mL. The thyroid function was normal. No autoimmune diseases and tumor diseases laboratory positive findings. The peak value of the insulin release test fell and the oral glucose tolerance test (OGTT) showed DM in Table 1. Diurnal rhythm changes of serum adrenocorticotropic hormone (ACTH), cortisol and thyroid function were nomal as shown in Table 2. Color Doppler prompted left atrial enlargement, diameter 38 mm; left kidney 140 mm × 58 mm × 75 mm, right kidney 138 mm × 56 mm × 77 mm.

**Table 1:** OGTT and insulin release test.

**Time (minute)**	**Before**	**30**	**60**	**120**	**180**
Blood glucose (mmol/L)	6.8	11.1	13.9	11.7	6.5
Insulin (μU/mL)	7.60	19.40	29.50	25.80	20.60

*Oral 50% glucose 150 mL (75 g glucose). The reference range of insulin base value in insulin release test is 2.2–11.6 μU/mL, and the detection method is chemiluminescence method*.

**Table 2:** Diurnal rhythm changes of serum ACTH and cortisol.

**Time**	**8:00**	**16:00**	**0:00**
ACTH (pg/mL)	10.60	<5.00	<5.00
Cortisol (pg/dL)	10.70	5.76	5.45

**Figure 2:**
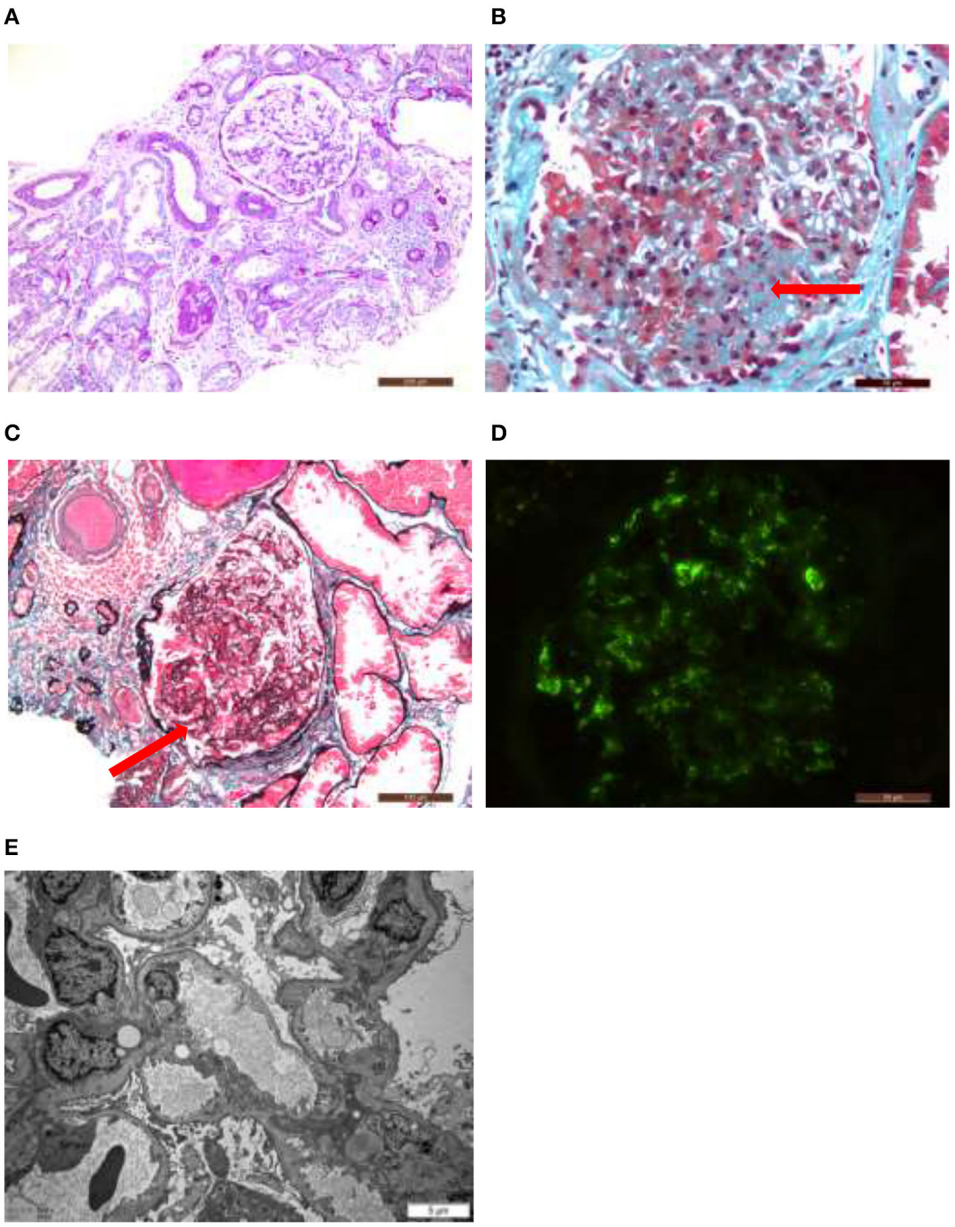
Hypertrophy of glomeruli, mild proliferation of mesangial cells, ischemic sclerotic glomeruli, focal atrophy of renal tubules, focal lymphocyte and monocyte infiltration with fibrosis in renal interstitium, and thickening of arteriolar wall **(A)**. Glomerular segmental sclerosis with podocyte hypertrophy, protein casts in renal tubule lumen. Focal atrophy, mononuclear cell infiltrate in renal interstitial with fibrosis **(B,C)**. Immunofluorescence study identified granular deposition of immunoglobulin A (±?+) and immunoglobulin M (++) in the mesangial area **(D)**. Electron microscopy showed that the foot processes of the glomerular visceral epithelial cells were widely fused, the segments of the basement membrane atrophy, and the deposition of electron density was occasionally seen in the mesangial area. The thickness of the basement membrane was 356.74 nm **(E)**.

After excluding contraindications, B-mode ultrasound guided renal biopsy was performed on January 15, 2018. Microscopic samples contained 24 glomeruli, including eight glomeruli with ischemic sclerosis, three glomeruli with ischemia and shrinkage, and one glomerular segmental sclerosis with glomerular epithelial cell proliferation. The rest were hypertrophy, mesangial cells proliferated slightly and mesangial matrix dilated mildly. Vacuolar and granular degeneration of renal tubular epithelial cells was observed. Small arteries had thickened wall and narrow lumen. The pathologic diagnosis was focal segmentular glomerular sclerosis [FSGS-NOS (not otherwise specified)] with ischemic renal injury and IgA deposition in the mesangial area (Classification based on Colombian typing in 2004) (Figure 2).

**Figure 3:**
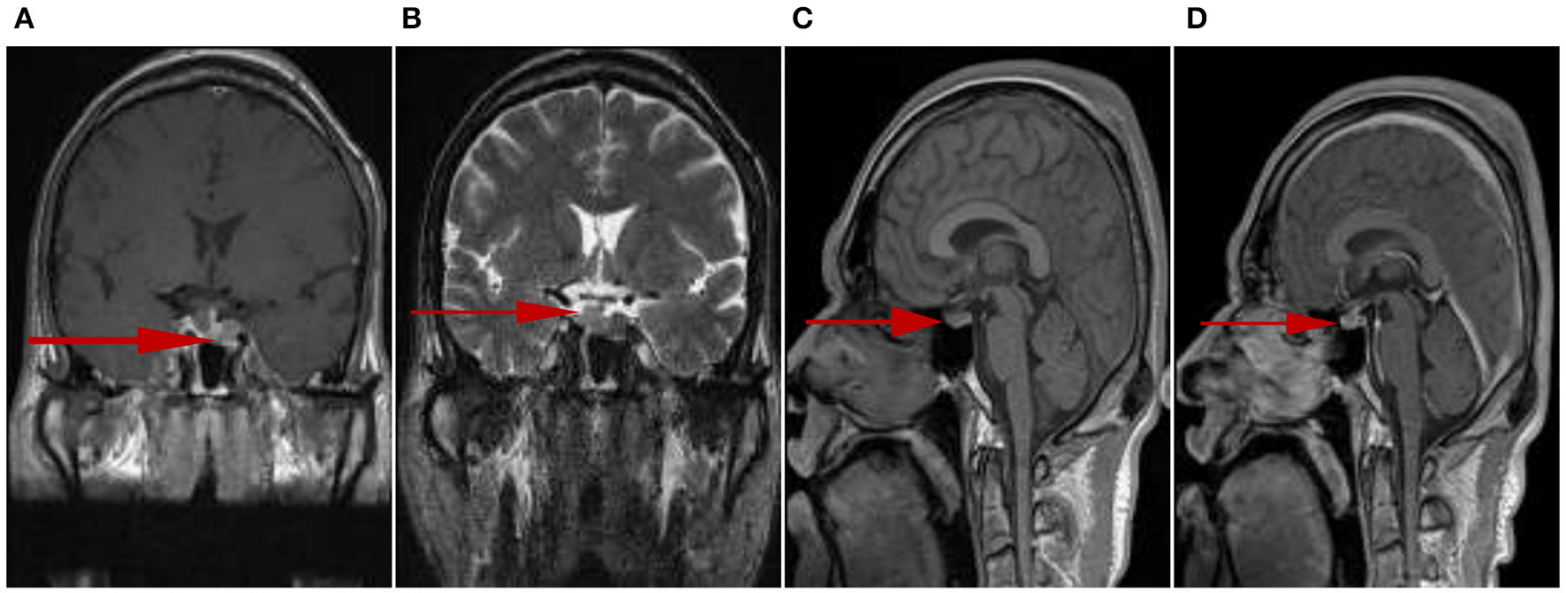
Irregular tumors could be observed in the sellar fossa, and the size was about 11 mm × 18 mm (upper and lower diameter × left and right diameter). T1 signal hyperintensity **(A)** and T2 signal heterogeneous intensity **(B)**, the lesion boundary was not clear. T1 signal isointensity could be seen on the left side of pituitary stalk, slightly high signal intensity on T1 could be seen on the right side of pituitary stalk **(C)**. The left side of pituitary stalk was enhanced, the range was 6 mm × 9 mm × 19 mm (left and right diameter × up and down diameter × front and rear diameter) **(D)**.

**Figure 4:**
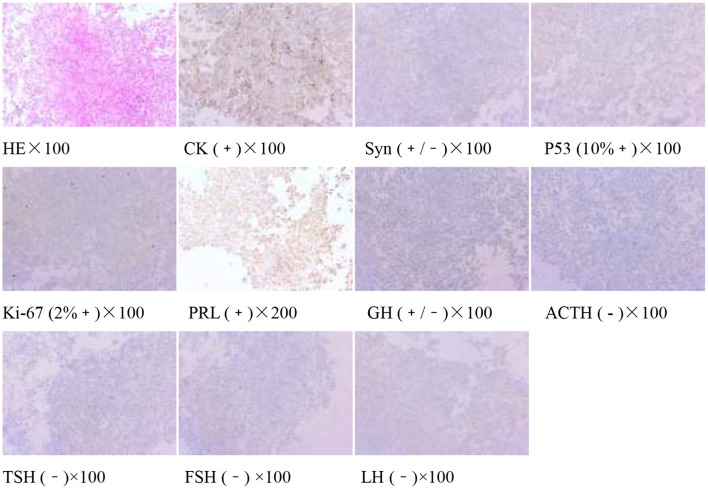
Pituitary adenoma (sellarr region) immunohistochemistry: CK (+), Syn (+/−), P53 (10%+), Ki-67 (2%+), PRL(+), GH (+/−), ACTH(−), TSH (−), FSH (−), and LH (−).

The pituitary enhanced magnetic resonance imaging was abnormal as shown in Figure 3. Transnasal space-occupying resection of the sellar region was performed on January 25, 2018. The pituitary adenomas was confirmed by pathology as shown in Figure 4.

**Figure 5:**
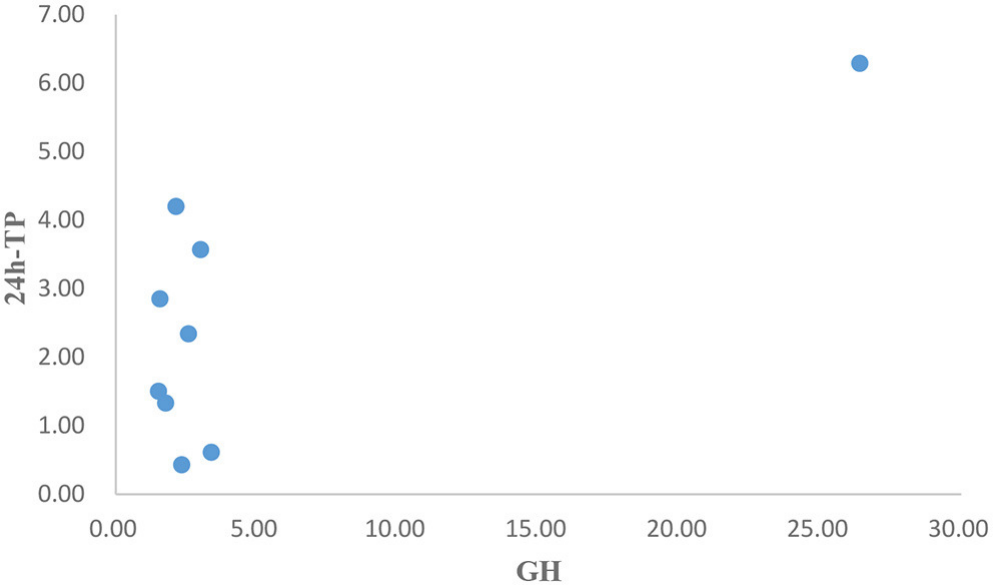
Scatter plot graph of GH and 24h-TP.

After operation, the patient received 50 mg/d of hydrocortisone intravenously for 3 days, followed by 40 mg/d for 3 days, 30 mg/d for 3 days, and 20 mg/d for 3 days. Then oral glucocorticoids 10 mg/d was used for hormone replacement therapy, combined with angiotensin receptor II antagonist to reduce glomerular hypertension. 1 month after operation, the level of GH rapidly decreased to 2.58 ng/mL, urinary protein decreased to 2.34 g/d, and nephropathy was partially relieved. In the next 4 months, the GH was in the normal range, but the urinary protein gradually increased to 4.2 g/d. Meanwhile, we improved gene detection and screened nephropathy-related genes in gene bank by high-throughput sequencing, including FSGS related genes ACTN4, ANLN, ARHGAP24, COL4A3, COL4A4, COQ2, COQ6, CUBN, INF2, LAMB2, LMX1B, NPHS1, NPHS2, NXF5, PDSS2, PLCE1, SCARB2, SMARCAL1, TRPC6, WDR73, WT1, etc. The patient's presentation was not explained by the gene variants associated with FSGS that were analyzed by sequencing. However, the FAN1 gene was detected, which was associated with karyomegalic interstitial nephritis, but renal biopsy did not support it. We tried to give glucocorticoids 20 mg/d combined with tacrolimus 1 mg/d and tripterygium wilfordii polyglycosides tablets 60 mg/d. The urinary protein decreased to 1.5 g/d again after 2 months. We gradually reduced the amount of glucocorticoids. When the urinary protein decreased to 0.43 g/d, he stopped taking both glucocorticoids and tacrolimus. After 8 months, the GH and IGF-1 were 1.56 ng/mL and 530.7 ng/mL, respectively. The urinary protein was 2.85 g/d. Color doppler examination showed that the left kidney was 124 mm × 70 mm × 65 mm, and the right kidney was 121 mm × 68 mm × 62 mm. Renal function of the patients was monitored in a stable state during the follow-up period. The clinical course of the patient before and after surgery is shown in Tables 3.1-3.3. Figure 5 is a scatter plot graph drawn with two sets of data, GH and 24h-TP. From the scatter plot graph, there is no obvious linear relationship between them, so it is not suitable to do linear correlation analysis. This may be related to the small sample size. Among the nine groups of data used to draw the scatter plot graph, only one set of data has a GH value that exceeds the normal range, while the other eight groups fluctuate within the normal range.

**Table 3.1:** The changes of ALB, 24h-TP, SCr, and eGFR.

**Time (month)**	0*	**1**	**2**	**5**	7***	**11**	**13**	**16**	**25**
ALB (g/L)	37.10	37.70	48.30	43.80	38.70	40.30	42.70	41.10	40.70
24h-TP (g)	6.29	2.34	3.57	4.20	1.50	1.33	0.61	0.43	2.85
SCr (μmol/L)	61.00	70.00	70.00	78.00	68.00	59.00	84.00	75.00	76.00
eGFR (mL/min/1.72m^2^)	111.47	105.34	105.34	100.05	106.60	112.21	92.93	100.95	100.42

**Table 3.2:** The changes of GH and IGF-1.

**Time (month)**	0*	0.5**	**1**	**2**	**5**	7***	**11**	**13**	**16**	**25**
GH (ng/mL)	26.40	6.44	2.58	3.01	2.14	1.52	1.77	3.39	2.34	1.56
IGF-1 (ng/mL)	1108.0	997.0	824.5	732.0	642.1	600.0	555.0	489.3	457.3	536.7

*The determination methods of GH and IGF-1 are chemiluminescence method, and their reference ranges are 0.06–5 ng/mL and 94–252 ng/mL, respectively*.

**Table 3.3:** The changes of blood pressure and heart rate.

**Time (month)**	0^*^	**2**	**5**	7^***^	**11**	**13**	**16**	**25**
Blood pressure (mmHg)	154/86	135/80	130/80	135/79	125/66	124/88	130/80	119/78
Heart rate (beats/minute)	86	86	80	68	68	88	72	74

* *The first day of admission*;

** *The third day after transnasal transsphenoidal space occupying resection*;

*** *The first reexamination after the addition of tacrolimus*.

## Discussion

In our case of acromegaly with FSGS, the patient had significant renal manifestations 10 years after the onset of acromegaly, indicating slow progression of renal lesions. After the surgery for pituitary adenomas, the urinary protein decreased significantly with the significant decrease of GH, and the nephropathy was partially relieved, but the level of IGF-1 was still significantly higher than the normal value. It can be inferred that the decrease of urinary protein is related to the decrease of GH, which is involved in the progress of FSGS. Nearly 2 years after operation, with the level of GH returned to normal, the size of kidney became smaller, but in the normal range, which suggests that high GH in acromegaly may be associated with renal hypertrophy. During the follow-up period, although the level of IGF-1 in the patient showed a downward trend, it was always higher than the normal level.

So far, three cases of FSGS complicated with acromegaly have been reported. In the first case reported in 1999, during the treatment of FSGS, repeated recurrence occurred with the reduction of glucocorticoids, but the risk of recurrence could be avoided by using low-dose glucocorticoids after pituitary microadenoma resection. It is suggested that GH is involved in the development of FSGS (7). In the second case reported in 2001, the levels of GH and IGF-1 returned to normal after resection of pituitary adenomas, but albuminuria continued. This case indicates that once FSGS is present in an acromegalic patient, cessation of GH overproduction may not be enough to reverse it (8). In the third case reported in 2019, there was no response to adequate glucocorticoids administration outside the hospital for 9 months. Nephropathy was partially relieved after subtotal resection of pituitary adenomas, and continued remission during the course of glucocorticoids reduction during follow-up (9). In addition, there are some cases that indirectly reflect the possible relationship between GH and FSGS. For example, after renal transplantation, child with growth retardation can be successfully treated with recombinant human growth hormone (rhGH). However, his renal function progressively deteriorated over a period of 32 months and ending up on dialysis (10). GH has been used by professional athletes and elite bodybuilders due to their enhancing effects on muscle growth. However, its side effects are serious and glomerular changes of FSGS have been reported in professional body-builders (11). Moreover, secondary FSGS is reported with endocrine disorders such as Cushing syndrome, Addison's disease, acromegaly and panhypopituarism, indicating hypothalamic-pituitary axis is also involved in glomerulosclerosis (12–14).

There has been a lot of research on the relationship between GH and kidney. GH receptor, IGF-1, IGF-1 receptor, and IGF binding protein genes are physiologically expressed in the adult kidney and vary according to the anatomy and functional segments of the nephron. However, when GH is hypersecretion, GH/IGF-1 axis increases glomerular filtration rate and kidney weight, causes glomerular hypertrophy, and leads to hyperphosphatemia, hypercalciuria, and sodium retention by affecting renal tubules (5). Now we can basically confirm the relationship between GH and FSGS, but whether GH is the cause of FSGS or merely accelerates the progress of FGGS, whether the pathological changes of FSGS can be reversed after removing the influence of GH remains to be further studied. The relations between the roles of GH and IGF-1 in promoting glomerulus hypertrophy and glomerulosclerosis are complicated. In transgenic mice, IGF-1 was found to be the cause of proliferation, not glomerulosclerosis (15). It was proved that the development of glomerulosclerosis in GH transgenic mice could be independent of IGF-1 (6). In most forms of FSGS, podocytes seem to be the central cells of injury (16). By activating recognized GH mediators (JAK2, STAT5, SH2-B, ERK1/2), podocytes activate GH receptors on the surface of podocytes to affect their cytoskeleton, which could have for consequence an increase in glomerular basement membrane pore size and eventually lead to the formation of microalbuminuria (5). But the role of IGF-1 in reducing urinary protein and delaying the progression of nephropathy is not clear.

Active acromegaly is often associated with changes in glucose homeostasis, such as insulin resistance, hyperinsulinemia, IGT or DM (17). Proteinuria levels in patients with IGT and DM are higher than in patients with normal glucose tolerance (18). In our case, the patient had a history of DM and the blood glucose control was poor before admission. The blood glucose was controlled during the follow-up period, which seems to support the relationship between urinary protein and changes in glucose homeostasis caused by acromegaly.

We considered the possibility of other causes for proteinuria including diabetic nephropathy (DN) and IgA nephropathy (IgAN). DN is a microvascular complication caused by long-term out of control of diabetes. In our case, the patient had a short history of diabetes, no thickening of basement membrane and no typical K-W nodule in renal biopsy. Therefore, the diagnosis of diabetic nephropathy is not considered. In addition, lesions morphologically identical with FSGS may appear in IgAN. Although IgA deposition was seen in immunofluorescence in the kidney pathology of this patient, it was weak, and there was no complement deposition, and there was no typical electron microscopic manifestation. From the clinical manifestation, the hematuria of the patient was not obvious, and no gene related to IgA nephropathy was found in gene screening. Therefore, we do not consider that IgAN is involved in the pathogenesis of this patient.

The diagnosis of FSGS is a descriptive pathological diagnosis, which in certain clinical situations (primary or idiopathic) becomes its own disease. The clinical diversity, histological diversity and non-specific morphological features of FSGS make the pathological diagnosis of FSGS complex and problematic (19). The FSGS-NOS is actually a kind of exclusion diagnosis, excluding perihilar, cell, tip and collapse variants, at least one glomerulus with segmental increase in matrix obliterating the capillary lumina and there may be segmental glomerular capillary wall (20).

Primary or idiopathic FSGS is an unknown circulatory factor that mediates abnormal glomerular permeability and podocyte injury and dedifferentiation. Secondary FSGS refers to the development of FSGS lesion as an adaptive phenomenon after the decrease of nephron mass, the direct toxicity of drug or virus infection or the healing of endothelial injury. Differential diagnosis of primary and secondary FSGS is important for treatment and prognosis. Distinguishing between primary FSGS and secondary FSGS is often difficult because of the considerable overlap in clinical and histological characteristics (21). Secondary FSGS tends to have proteinuria in the range of subnephropathy without edema and hypoproteinemia, and nephrotic range proteinuria will develop over time. Pathologically, the disappearance of the foot process of primary FSGS is diffuse (3). The purpose of FSGS treatment is to reduce urinary protein and delay the progression of nephropathy. Primary FSGS is mainly treated with glucocorticoids. Patients with FSGS who are resistant to or dependent on glucocorticoids can be treated with immunosuppressant. Studies have shown that the use of calcineurin inhibitors and/or glucocorticoids as part of immunosuppressive therapy in the early immunosuppressive regimen of primary FSGS is associated with improving renal prognosis (22). Patients with secondary FSGS should actively look for the causes of FSGS. Whether the causes need immunosuppressive therapy is still an uncertain question (21).

This patient had no edema and hypoproteinemia at admission and had proteinuria within the range of nephropathy. Renal biopsy showed extensive fusion of foot processes. After operation, the patient received glucocorticoids replacement therapy and angiotensin II receptor antagonist. Five months after operation, urinary protein increased again, GH was still in the normal range, and the angiotensin II receptor antagonist we used also had an independent role in lowering urine protein, which was similar to the previous case report in 2001. On the one hand, we considered that the pathological changes of kidney caused by GH were not completely reversible, on the other hand, we speculated that other factors were involved. With the re-elevation of urinary protein, we chose to adjust the treatment regimen by adding immunosuppressants. High doses of glucocorticoids may affect pituitary function, it can directly inhibit the secretion of pituitary GH, gonadotropin and thyrotropin (23). Then we gave low-dose glucocorticoids combined with tacrolimus, urinary protein decreased significantly and the renal function was always at the normal level. The reason why small doses of glucocorticoids combined with tacrolimus are effective may be that immune factors are involved in the pathogenesis.

In conclusion, we believe in this case that GH is partially involved in the progression of FSGS. Adenoma resection and high GH deprivation are the major to the improvement of nephropathy in this patient. Immune factors may be involved in the patient and respond to low-dose glucocorticoids combined with tacrolimus therapy. Therefore, it is suggested that clinicians pay attention to the renal lesions of patients with GH hypersecretion as early as possible, and early treatment may delay the progression of nephropathy. Meanwhile, for patients with FSGS, the involvement of GH in the search for the cause may also be considered.

## Data Availability Statement

The original contributions presented in the study are included in the article/supplementary material, further inquiries can be directed to the corresponding author/s.

## Ethics Statement

The studies involving human participants were reviewed and approved by The First Affiliated Hospital of Zhengzhou University. The patients/participants provided their written informed consent to participate in this study.

## Author Contributions

All authors listed have made a substantial, direct and intellectual contribution to the work, and approved it for publication.

## Conflict of Interest

The authors declare that the research was conducted in the absence of any commercial or financial relationships that could be construed as a potential conflict of interest.
